# Clinical experience of pelvic radiotherapy or chemoradiotherapy for postoperative uterine cervical cancer using intensity-modulated radiation therapy

**DOI:** 10.1093/jrr/rraa004

**Published:** 2020-02-26

**Authors:** Takaya Yamamoto, Rei Umezawa, Hideki Tokunaga, Masaki Kubozono, Maiko Kozumi, Noriyoshi Takahashi, Haruo Matsushita, Noriyuki Kadoya, Kengo Ito, Kiyokazu Sato, Keita Tsuji, Muneaki Shimada, Keiichi Jingu

**Affiliations:** 1 Department of Radiation Oncology, Tohoku University Graduate School of Medicine, Sendai, Japan; 2 Department of Obstetrics and Gynecology, Tohoku University Graduate School of Medicine, Sendai, Japan; 3 Department of Radiation Oncology, Miyagi Cancer Center, Natori, Japan; 4 Division of Radiology, Tohoku Medical and Pharmaceutical University, Sendai, Japan; 5 Radiation Technology, Tohoku University Hospital, Sendai, Japan

**Keywords:** postoperative, pelvic radiotherapy, uterine cervical cancer, intensity-modulated radiotherapy, VMAT, IMRT

## Abstract

The purpose of this study was to reveal treatment outcomes and toxicity after pelvic intensity-modulated radiotherapy (IMRT) for postoperative uterine cervical cancer of Japanese patients. Consecutive patients who were treated with pelvic IMRT for postoperative cervical cancer in our institute were retrospectively analyzed. Relapse-free survival (RFS) and overall survival (OS) were calculated using the Kaplan–Meier estimator, and log-rank tests were used to compare differences. From the database, 62 patients were identified. The pathology was squamous cell carcinoma in 44 patients and other pathology in 18 patients. Of the 62 patients, 35 had high-risk prognostic factors and 27 patients had intermediate-risk prognostic factors. The prescribed radiation doses were 50 Gy in 25 fractions for 58 patients and 50.4 Gy in 28 fractions for 4 patients. One patient received a vaginal cuff boost. Chemotherapy was administered in 36 patients. During the median follow-up period of 50.9 months, there was no locoregional failure. Six patients in the high-risk group relapsed, but none of the patients in the intermediate-risk group relapsed (*P* = 0.02). The 3-year OS and RFS rates were 98.2% and 90.9%, respectively. Significant factors related to RFS were squamous cell carcinoma pathology (*P* = 0.02), pathological T stage (*P* = 0.04), surgical margin status (*P* < 0.01) and multiple lymph nodes metastases (*P* < 0.01). Grade 3 or more toxicity occurred in 6 patients. Four patients had obstruction of the intestine, and 2 patients had stenosis of the urinary tract. In clinical practice, the use of pelvic IMRT for postoperative cervical cancer of Japanese patients showed a low rate of toxicity without decreasing the efficacy.

## Introduction

Definitive treatment for early-stage uterine cervical cancer is surgery or radiotherapy with or without chemotherapy. In surgical treatments, adjuvant treatment for patients who have prognostic risk factors after radical hysterectomy has been recommended [1, 2]. Adjuvant treatment is also recommended in Japanese treatment guidelines for uterine cervical cancer, but adjuvant treatment has not always been used in clinical practice in Japan. A clinical survey conducted in 2010 and 2014 in Japan showed that >80% of institutions in Japan chose surgery as the primary therapy for Stage IB1 and IIA1 tumors, and chemotherapy alone as an adjuvant treatment was considered one of the key options in 72% of institutions in expectation of the reduction of complications caused by radiotherapy [3–5]. Since then, prospective trials have followed the clinical practice [6]. However, radiotherapy now has reduced complications through the use of the intensity-modulated radiation therapy (IMRT) technique [7, 8]. It has been reported that IMRT dramatically reduced the incidence of complications compared with the level when 3D radiotherapy (3DRT) was used in postoperative uterine cancer patients. IMRT decreased the 3-year cumulative incidence of Grade 2 or higher gastrointestinal toxicity from 45% to 3% [9]. It has also been reported that IMRT might contribute more than 3DRT to a better quality of life. [10].

In spite of these progressions, gynecological oncologists in Japan tend to shun radiotherapy. However, pelvic IMRT has been positively used in a postoperative setting in our institute in collaboration with gynecological oncologists. The aim of this study was to reveal the treatment outcomes and toxicities after pelvic radiotherapy for postoperative cervical cancer in clinical practice in an academic environment. It was predicted that the results would provide important data, especially for the benefit of Japanese patients.

## Materials and Methods

### Eligibility and informed consent

Patients who received pelvic radiotherapy or chemoradiotherapy involving IMRT for postoperative uterine cervical cancer in our hospital were included in this study. Patients who had been treated with only cervical conization, or patients who had already relapsed before radiotherapy, were not considered to be in a postoperative condition. To avoid selection bias, all of the remaining patients, i.e. consecutive patients who received pelvic IMRT for postoperative cervical cancer, were enrolled in this retrospective study. This study was approved by the Ethical Committee of Tohoku University Hospital (No. 2019–1-648). Informed consent was waived due to the retrospective nature of this study. All patients were guaranteed the chance to restrict their data use in this study by giving information on this study via the Internet. Furthermore, written informed consent as a part of general consent for utilization of treatment data in future retrospective studies was obtained from all patients who have been treated after April 2016.

### CT simulation and radiotherapy

Before CT simulation, two small cotton balls were inserted into the vaginal cuff of each patient. Each patient was immobilized in the supine position with a vacuum cushion (VacQfix Cushion, Qfix, Avondale PA, USA). Treatment planning CT scans at intervals of 2.0–2.5 mm were performed in a full bladder state, and CT scans in a empty bladder were also performed to compensate for internal organ motion. The nodal clinical target volume (CTV) was contoured on the basis of the Japan Clinical Oncology Group (JCOG) consensus-based guideline [11]. The vaginal and parametrial CTV was contoured 1.0–1.5 cm superiorly from the inserted cotton and 3–4 cm inferiorly or at the bottom level of the obturator foramen. The vaginal and parametrial internal target volume (ITV) was created from the vaginal and parametrial CTV and empty bladder CT scans. The planning target volume (PTV) was created by expanding 0.7 cm from the nodal CTV and 1.0 cm from the vaginal CTV in all directions. In principal, 50 Gy in 25 fractions covering 90% of the PTV were prescribed, and radiotherapy was performed under the image guidance using a linear accelerator equipped with cone-beam CT. IMRT was performed using the volumetric-modulated arc therapy (VMAT) technique, and coplanar multi-arc beams with 10 MV or 15 MV photons were used. Dose distribution in the PTV also needed the maximum dose to be < 115% of the prescribed dose, < 1% of the PTV to receive >110% of the prescribed dose, and >99% of the PTV to receive >91% of the prescribed dose. An organ at risk was contoured on the basis of the ‘Female RTOG Normal Pelvis Atlas’ provided by the Radiation Therapy Oncology Group (RTOG). Normal tissue dose constraints were as follows: < 35% of the bladder received <45 Gy; <80% and <35% of the rectum received <40 Gy and <50 Gy, respectively; <30% and maximum dose of the bowel bag received <40 Gy and <110% of the prescribed dose, respectively; <37% and <90% of the pelvic bone received <40 Gy and <10 Gy, respectively; and <40% of the femoral heads received <30 Gy. When there were transposed ovaries, the mean dose each ovary received was <3 Gy or <7 Gy, which were the desired value and tolerant value, respectively. A vaginal cuff boost using intracavitary brachytherapy was performed when there was a residue of invasive cancer: typically, 12 Gy in 2 fractions prescribed at 5 mm beyond the vaginal mucosa.

### Postoperative risk factors and chemotherapy

Patients in the high-risk prognostic factor group were defined as patients with at least one of the high-risk prognostic factors, including positive pelvic lymph nodes, parametrial extension, and positive surgical margins [12]. An additional risk factor was metastasis to the ovary. Patients in the intermediate-risk prognostic factor group were defined as patients with at least one of the intermediate-risk prognostic factors without high-risk prognostic factors. The intermediate-risk prognostic factors were: greater than one-second stromal invasion, lymphovascular space invasion, and cervical tumor diameters of >4 cm.

Chemotherapy was performed in patients with high-risk prognostic factors. On the other hand, patients in the intermediate-risk group were treated with radiotherapy alone. When the pathology was squamous cell carcinoma, adenocarcinoma, or adenosquamous carcinoma, concurrent chemotherapy was platinum monochemotherapy. In our hospital, weekly cisplatin (40 mg/m^2^) was administered for up to five cycles, and if the patients was intolerant to cisplatin, weekly nedaplatin was used as an alternative [13]. Adjuvant chemotherapy was not administered. When the pathology was neuroendocrine tumors (typically small-cell carcinoma or large-cell neuroendocrine carcinoma), concurrent and adjuvant chemotherapy was performed, with three cycles of a regimen consisting of vincristine, adriamycin and cyclophosphamide alternating with cisplatin and etoposide (VAC/PE) [14].

**Table 1:** Patient demographics at the time of radiotherapy

Characteristics		Number (%)
Patients		62
Age, years	Median, range	45, 29–79
Period of surgery	September 2013 – February 2019	
Surgery type	Radical hysterectomy	58 (93.5)
	Total hysterectomy	4 (6.4)
Pathology	Squamous cell carcinoma	44 (70.9)
	Adenocarcinoma	11 (17.7)
	Mixed	1 (1.6)
	Adenosquamous carcinoma	3 (4.8)
	Neuroendocrine carcinoma	3 (4.8)
Clinical stage (FIGO 2008)	IB1	39 (62.9)
	IB2	9 (14.5)
	IIA1	4 (6.4)
	IIA2	3 (4.8)
	IIB	7 (11.2)
Pathological T stage (UICC 8th edn)	pT1b1	32 (51.6)
	pT1b2	10 (16.1)
	pT2a1	2 (3.2)
	pT2a2	3 (4.8)
	pT2b	15 (24.1)
Parametrial extension	Yes	15 (24.1)
	No	47 (75.8)
Nodal involvement	None	34 (54.8)
	1	7 (11.2)
	≥2	21 (33.8)
Surgical margin (microscopic positive margin)	Yes	2 (3.2)
	No	60 (96.7)
Stromal invasion	≥1/2	45 (72.5)
	<1/2	17 (27.4)
Lymphovascular space invasion	Yes	34 (54.8)
	No	28 (45.1)
Tumor diameter, cm	Median, range	3.0, 0.5–7.4
Prognostic risk group	High-risk group	35 (56.4)
	Intermediate-risk group	27 (43.5)
Ovarian transposition	Yes	19 (30.6)
	No	43 (69.3)
Interval between surgery and radiotherapy, days	Median, range	51 (27–109)

### Outcome and toxicity assessment

Locoregional relapse was defined as any intrapelvic recurrence or metastasis excluding pelvic bone metastasis and peritoneal dissemination, and locoregional control was defined as freedom from locoregional relapse. Relapse-free survival (RFS) was defined as freedom from any metastases, any recurrences, dissemination or death. Overall survival (OS) was defined as freedom from death by all causes. Toxicity was assessed from the day radiotherapy was started. Acute toxicity of (chemo)radiation was assessed according to the National Cancer Institute Common Terminology Criteria for Adverse Events version 5.0 translated by the Japanese Clinical Oncology Group (CTCAE v5.0-JCOG). Late toxicity was graded according to the RTOG Late Radiation Morbidity Scoring Scheme [15]. Urinary tract obstruction and lymph edema were assessed using CTCAE v5.0-JCOG because there was no adequate definition in the RTOG scheme.

### Statistical analysis

The time to an event was calculated from the day of definitive surgery to the day that an event was confirmed. The cumulative RFS rate and the OS rate were calculated using the Kaplan–Meier estimator, and the log-rank test was used to compare differences. A *P* value less than 0.05 was defined as significant. EZR version 1.37 (Saitama Medical Center, Jichi Medical University, Saitama, Japan), a modified version of R commander (R Foundation for Statistical Computing, Vienna, Austria), was used for analyses [16].

## Results

### Patients

A total of 62 patients were identified from our database. Patient demographics at the time of radiotherapy are summarized in Table 1. Of the 62 patients, 58 received radical hysterectomy and 4 received total hysterectomy; 19 patients aged from 29 to 43 years received ovarian transposition. There was no macroscopic residual case, and only two cases showed a microscopic positive surgical margin, including one case of carcinoma *in situ*. Of the 62 patients, 35 had high-risk prognostic factors and 27 had only intermediate-risk factors.

The results of radiotherapy and chemoradiotherapy are summarized in Table 2. The prescribed radiation doses were 50 Gy in 25 fractions in 58 patients, or 50.4 Gy in 28 fractions in 4 patients. One patient who had a positive surgical margin received a vaginal cuff boost, using intracavitary brachytherapy with 12 Gy in 2 fractions. Chemotherapy was administered in 36 patients, including 2 of the 4 patients who received only total hysterectomy in the intermediate-risk group. One patient in the high-risk group who had one high-risk factor (a positive surgical margin) did not receive chemotherapy. All patients completed the planned radiotherapy.

**Table 2:** Chemoradiotherapy characteristics and patterns of recurrence

Characteristics		Number (%)
Radiotherapy	50 Gy/25fr	58 (93.5)
	50.4 Gy/28fr	4 (6.4)
Intracavitary brachytherapy boost	12 Gy/2fr	1 (1.6)
Chemotherapy	Yes	36 (58.0)
–Weekly platinum		32
	Cisplatin	30 (93.7)
	Nedaplatin	2 (6.2)
–No. of cycles received	3	1 (3.1)
	4	6 (18.7)
	5	24 (75.0)
	6	1 (3.1)
Patterns of recurrence	Lung	2
	Liver	1
	Lymph nodes	1
	Lymph nodes and bone	1
	Peritoneal dissemination and spleen	1

### Treatment outcomes

**Fig. 1.**
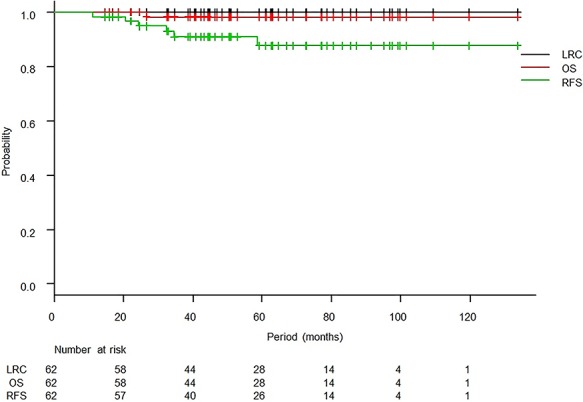
Kaplan–Meier curves of locoregional control (LRC), overall survival (OS) and relapse-free survival (RFS).

**Fig. 2.**
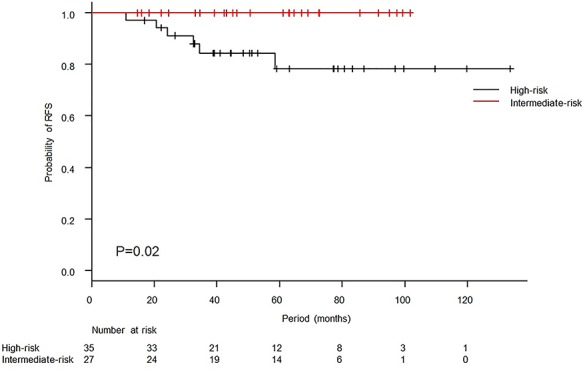
Kaplan–Meier curves of relapse-free survival (RFS) according to prognostic risk factors. The 3-year RFS rates in the intermediate-risk and high-risk groups were 100.0% and 84.3%, respectively. The difference showed significance (*P* = 0.02, log-rank test).

The median follow-up period was 50.9 months (range, 14.6–133.7 months), and one patient died of primary disease. There was no case of locoregional relapse. Six patients relapsed, and all of those patients were in the high-risk group. Initially relapsed sites are summarized in Table 2. Two cases developed lymph nodes recurrence, and both cases developed simultaneously para-aortic lymph nodes and supraclavicular lymph nodes recurrence. The 3-year OS and RFS rates were 98.2% [95% confidence interval (95% CI): 87.8–99.7%] and 90.9% (95% CI: 79.5–96.1%), respectively (Fig. 1). The 3-year RFS rates in the intermediate-risk and high-risk groups were 100.0% (95% CI: 100.0–100.0%) and 84.3% (95% CI: 66.2–93.2%), respectively, and the difference was significant (*P* = 0.02, Fig. 2). Variables of clinical and pathological findings were also compared using log-rank tests (Table 3). As a result, variables that were significant factors for RFS were pathology (squamous cell carcinoma vs others, *P* = 0.02), pT1 vs pT2 (*P* = 0.04), surgical margin status (*P* < 0.01) and pathological multiple lymph nodes metastases (*P* < 0.01). Ovarian function was maintained in 8 of 19 patients (5 of 11 patients who received chemoradiotherapy and 3 of 8 patients who received radiotherapy alone) who received ovarian transposition with a mean irradiated ovarian dose of 4.3 ± 2.0 Gy.

**Table 3:** Results of log-rank tests for RFS

Variables	No.	3-year RFS	*P* value
Age, years			
≤45	30	91.8	
>45	32	90.2	0.49
Surgery type			
Radical hysterectomy	58	90.4	
Total hysterectomy	4	100.0	0.53
Pathology			
Squamous cell carcinoma	44	95.1	
Others	18	80.8	0.02
Pathological T stage			
pT1	42	97.1	
pT2	20	78.5	0.04
Parametrial extension			
Yes	15	78.8	
No	47	95.0	0.12
Tumor diameter, cm			
≤4	50	88.6	
>4	12	100.0	0.20
Surgical margin (microscopic positive)			
Yes	2	Not reached	
No	60	92.4	<0.01
Pathological N stage			
pN0	34	96.2	
pN1	28	85.4	0.05
Nodal involvement			
0–1	41	97.0	
≥2	21	80.4	<0.01
Lymphovascular space invasion			
Yes	34	90.6	
No	28	91.3	0.55
Stromal invasion			
<1/2	17	93.8	
≥1/2	45	89.9	0.53

RFS = relapse-free survival, No. = number of patients.

### Toxicities

**Fig. 3.**
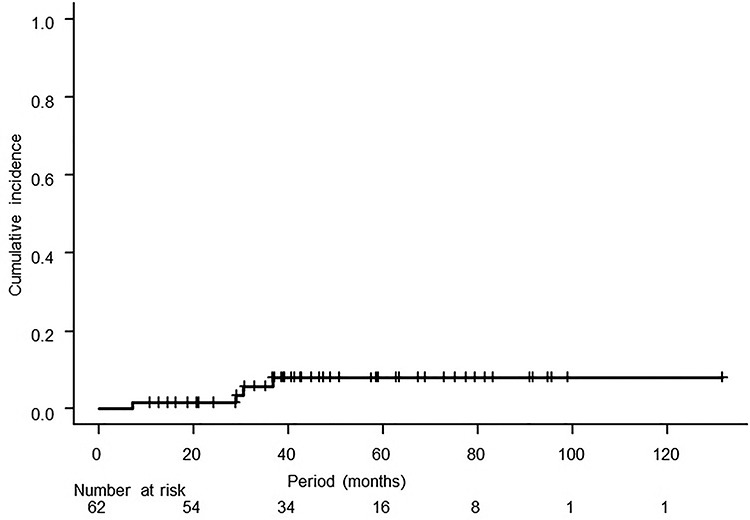
Cumulative incidence of Grade 3 or higher gastrointestinal toxicity starting from the first day of radiotherapy. The 3-year cumulative incidence was 5.8% (95% CI: 0.0–12.0%).

Dose calculation results of parameters for an organ at risk are shown in Table 4, and acute and late toxicities are summarized in Table 5. Grade 3 or more late toxicities occurred in 6 patients. Four patients had obstruction of the intestine, and perforation of the intestine occurred in one of those patients. The other two patients had urinary tract stenosis, which developed into severe hydronephrosis. Repeated admission to the hospital because of obstruction of the intestine was needed in four patients: three times in one patient, five times in one patient and six times in two patients. The 3-year cumulative incidence of Grade 3 or higher gastrointestinal toxicity was 5.8% (95% CI: 0.0–12.0%, Fig. 3).

**Table 4:** Dose calculation results of parameters for organ at risk

Structure	Median	Interquartile range	Range
Bladder			
Percentage of patients who received 45 Gy or more	42.0	34.2–52.8	24.7–81.9
Bowel bag			
Maximum dose (Gy)	54.4	54.0–54.7	52.6–55.1
Percentage of patients who received 40 Gy or more	28.7	25.3–31.9	12.6–54.7
Rectum			
Percentage of patients who received 40 Gy or more	65.8	58.3–73.7	36.8–97.3
Pelvic bone			
Percentage of patients who received 40 Gy or more	27.8	24.1–31.0	15.2–42.0
Percentage of patients who received 10 Gy or more	95.1	90.0–99.0	81.7–100.0
Ovaries (19 patients)			
Mean ovarian dose (Gy)	4.8	1.9–5.9	1.0–7.5

**Table 5:** Toxicities

Type of toxicity	Grade 2 or more	Grade 3 or more
Acute toxicities of chemoradiation		
Gastrointestinal	21 (69.0)	7 (11.2)
Hematologic	31 (86.1)	20 (55.5)
Acute toxicities of radiation		
Gastrointestinal	10 (16.1)	
Hematologic	10 (16.1)	
Late toxicities		
Bowel obstruction	8 (12.9)	4 (6.4)
Urinary tract		2 (3.2)
Lymph edema	3 (4.6)	

There was no Grade 2 or more toxicity regarding bowel bleeding and bladder. Data are expressed as number (%).

## Discussion

Although this study was a retrospective study conducted in a single institute in an academic environment, the results were meaningful, especially for Japanese patients. They accurately reflected outcomes in clinical practice owing to the inclusion of all patients who received pelvic IMRT for postoperative uterine cervical cancer in our institute. Pelvic IMRT for postoperative cervical cancer showed a low rate of toxicity without decreasing the efficacy; on the contrary, the result showed perfect locoregional control, and the locoregional control clearly contributed to excellent RFS. Pelvic IMRT might not only enable reduction of complications but also improve treatment outcomes compared with those in previous studies [5, 12, 17]. There are several possible reasons for the different results. First, the vaginal cuff was carefully targeted using cotton balls and cautiously irradiated by using cone-beam CT image guidance. Furthermore, cone-beam CT provided information about changes of ascites and body contour for considering replanning. Third, the JCOG consensus-based guideline for nodal CTV was described in detail; therefore, the nodal target was created larger than that based on the RTOG guideline [11]. Finally, the doses prescribed covered 90% of the PTV, greatly reducing the possibility of an under-dose area within the PTV.

Although there were good results for RFS in this study, the possible prognostic factors for RFS were almost the same as those previously reported [18]. Pelvic lymph node involvement was regarded as a high-risk factor, but the number of positive lymph nodes might also be important [5, 12, 19, 20]. Patients with squamous cell carcinoma also showed better RFS in several studies [5, 21, 22]. The emergence of these factors, despite sophisticated radiation techniques, might suggest the need for reinforcement of concurrent or adjuvant chemotherapy.

Ovarian function was maintained in only 8 of the 19 patients, and this result is not satisfactory [23]. Suppression of ovarian function is caused by chemotherapy and radiotherapy, especially by exposure of the ovaries to high radiation doses. The ovary is very radiosensitive, and strict restriction of the mean ovarian dose to 2.5–3.4 Gy in one study resulted in the maintenance of ovarian function in 5 (71%) of 7 patients [24]. Transposition of the ovaries out of the irradiation field is important for reducing ovarian doses. Four patients in that study had ovarian transposition at 3–3.5 cm above the level of L5–S1, and 1 patient had ovarian transposition at 1.5 cm from the radiation field edge [24]. In the present study, the mean ovarian doses were 1.5 ± 0.54 Gy when the ovaries were located above the PTV and 5.3 ± 0.32 Gy when the ovaries were located at the same level as the PTV (data not shown in the Results section). When the ovaries are located above the PTV, the ovaries are affected only by secondary radiation doses from IMRT and cone-beam CT with or without chemotherapy. On the other hand, when the ovaries are located at the same level as the PTV, the ovaries are affected by not only scattering but also by direct radiation of cone-beam CT, IMRT and leakage doses from the multileaf collimator. In such cases, the usual IMRT (VMAT) technique of strict dose constraints and the set of beam avoidance sector where the beam was turned off during VMAT were unsatisfactory, and the use of a special technique such as the restricted field technique might therefore be desirable [25].

The incidence of bowel obstruction was comparable with or lower than that in previous studies [5, 26]. According to a nationwide cohort study in Japan, Grade 3–4 bowel obstruction was observed in 7.4% of patients who received postoperative pelvic radiotherapy alone, 9.7% of patients who received postoperative pelvic chemoradiotherapy and 3.2% of patients who received adjuvant chemotherapy alone [27]. In this study, Grade 3–4 bowel obstruction occurred in 4 (6.4%) of the patients, and those 4 patients required repeated admission because of bowel obstruction. The rate of bowel obstruction was relatively low, but might not be considered satisfactory, compared with the rate after adjuvant chemotherapy alone. These are reasons why many gynecological oncologists shun radiotherapy. Since internal motion of the postoperative bowel is thought to be restricted because of postoperative adhesion, the definition of the bowel bag, dose constraints for the bowel and dose prescription should be reconsidered.

There are several limitations in the current study. This study was a retrospective single-institute study with a limited sample size. Because only six patients relapsed, multivariate analysis was not performed. Some heterogeneity of treatment (either surgery or radiotherapy) was seen. The indication for radiotherapy in patients with intermediate-risk factors, and the definition of intermediate-risk factors, especially the extent of stromal invasion, were controversial.

In conclusion, pelvic IMRT for postoperative cervical cancer of Japanese patients showed a low rate of late toxicities, without decreasing the efficacy, and these results were comparable with previous findings.
